# Single-cell electro-mechanical shear flow deformability cytometry

**DOI:** 10.1038/s41378-024-00810-5

**Published:** 2024-11-22

**Authors:** Junyu Chen, Xueping Zou, Daniel C. Spencer, Hywel Morgan

**Affiliations:** School of Electronics and Computer Science, and Institute for Life Sciences, University of Southampton, Southampton, SO17 1BJ UK

**Keywords:** Engineering, Physics

## Abstract

The complex structural and molecular features of a cell lead to a set of specific dielectric and mechanical properties which can serve as intrinsic phenotypic markers that enable different cell populations to be characterised and distinguished. We have developed a microfluidic technique that exploits non-contact shear flow deformability cytometry to simultaneously characterise both the electrical and mechanical properties of single cells at high speed. Cells flow along a microchannel and are deformed (elongated) to different degrees by the shear force created by a viscoelastic fluid and channel wall. The electrical impedance of each cell is measured using sets of integrated microelectrodes along two orthogonal axes to determine the shape change and thus the electrical deformability, together with cell dielectric properties. The system performance was evaluated by measuring the electro-mechanical properties of cells treated in different ways, including osmotic shock, glutaraldehyde cross-linking and cytoskeletal disruption with Cytochalasin D and Latrunculin B. To confirm the accuracy of the system images of deformed cells were also captured using a camera. Correlation between the optical deformability and the electrical deformability is excellent. This novel cytometer has a throughput of ~100 cells s^—1^ is simple, does not use sheath flow or require high speed optical imaging.

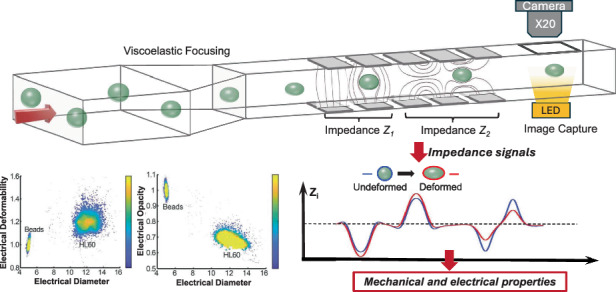

## Introduction

Label-free single cell analysis methods are of growing interest because they provide direct measurement of phenotype, particularly mechanical or electrical properties. The mechanical properties which manifest through cell deformability are closely related to intracellular structure, particularly of the cytoskeleton and nucleus^1^. Many different properties are linked to cell deformation, including cell cycle^2,3^, cancer^4–6^ immune cell activation^7,8^ and stem cell differentiation^6,9^. Single-cell mechanical phenotyping can be performed direct from biopsy samples in order to determine inflammation and discriminate healthy from tumour tissue^10^.

Single-cell mechanical analysis has been performed using several different techniques^11^, including AFM^12,13^, acoustic scattering^14^, optical stretching^15^, and micropipette aspiration^16^. However, these methods are not high throughput and can be technically demanding; to address this microfluidic single-cell cytometric methods have been developed^1^. One technique is contact-based deformability cytometry (cDC), where cell stiffness is determined from the transit time as cells squeeze through a narrow constriction. The transit time is measured using techniques such as optical imaging^17–19^ resonating cantilever methods (which can also determine cell buoyant mass)^20^, or electrical resistance/impedance methods^21–24^, including electrical node pore sensing^25^. Constriction-based methods have also been developed to characterise both the electrical and mechanical properties of single cells^26^ including extracting the Young’s modulus, fluidity and capacitance of single cells^27,28^. Recently an optical stretcher has been coupled with 3D electrorotation electrodes to perform multiparameter characterisation of single cells^29^.

Contact based methods are generally low throughput, influenced by clogging of the channel and measure a narrow range of cell sizes. Furthermore, cell transit time can be influenced not only by deformability but also by cell volume and membrane-wall friction and interactions.

To address these limitations noncontact analysis methods have been developed where a hydrodynamic flow induces a shape change in the cell, eliminating interaction between the cell and the channel wall. High-speed cameras and image processing measure cell shape from which cell deformability is inferred. Shear flow deformability cytometry (sDC) uses velocity gradients to generate shear stress in a microchannel slightly larger than the cell to deform the cell into a bullet shape^30,31^. Shear forces dominate, and this technique is mostly sensitive to changes in the cytoskeleton but not the nuclear structure.

Extensional flow deformability cytometry (xDC) uses fluid-induced stress to deform cells at a stagnation point, normally with a cross shaped microfluidic channel^5,6,32^. Inertial forces induce changes in a few micro-seconds meaning that analysis rates exceed 1000 cells per second (high *Re*). The dominant compressional force from the fluid inertia deforms the cells. Guillou et al.^33^ used an extensional flow device but at much lower Re numbers where shear forces dominate and observed changes due to actin destabilisation. Armistead et al.^34^ described a device that covers both flow regimes from high to low strain in both shear and inertia dominant regimes and showed that different regimes probe different aspects of the cell structure, demonstrating that the shear-dominant, low-strain regime is most sensitive to cytoskeletal changes. The three different techniques were recently compared^35^, confirming that the higher strain rate of xDC makes measurement of cytoskeletal changes (actin destabilisation) challenging, possibly due to cytoskeletal fluidization^34^.

Analogous to the field of cell mechanics, probing cell phenotypic electrical properties has been of interest for many years. Traditionally cells were analysed in suspension, but microfluidic high-speed single cell impedance methods allowing heterogeneity in populations to be identified. Cell electrical properties reflect fundamental cellular physiology, for example cell cycle^36^, activation/function^37^, cytoskeleton^38^; and single-cell impedance analysis has been used for tumour cell stratification/separation^39,40^, leukocyte analysis^41^ and to identify parasite invasion^42^. Single-cell impedance analysis is usually performed using microfluidic devices with micro-electrodes that measure the impedance of a microchannel as cells transit between successive pairs of electrodes^43,44^. Traditionally measurements are made at two AC frequencies, typically a lower frequency (high kHz) to measure cell volume and a second higher frequency to measure cell membrane properties. The ratio of these two impedances is termed the electrical opacity^44^ and indirectly characterises the cell membrane. Single cell multi-frequency measurements have also been demonstrated providing a complete electrical phenotype by fitting data to a lumped-parameter model^45^.

Given the growing interest in label-free techniques, and their translational potential for diagnosing disease, techniques that simultaneously measure both the mechanical and electrical properties of cells may provide important insights into cell behaviour and disease pathology. Recently a non-contact impedance-based deformability cytometer was described^46^. This system measures cell deformability using electrical rather than optical methods and measures both the electrical and mechanical properties of single cells at moderate throughput (10-20 cells per second). Viscoelastic-inertial sheath flow was used to focus cells into a narrow stream that flows through a cross-junction where cells are deformed due to pinching from sheath fluids. In this system shear force dominates over the compressive force. The change in cell shape was determined by comparing the impedance signal before and after a cell passes along the cross-shaped microchannel. Size, deformability and electrical opacity of neutrophils was measured, demonstrating changes upon activation. Reale et al.^47^ used extensional flow created with a hyperbolic channel to induce cell deformation. Planar microelectrodes at a cross junction measure the orthogonal and lateral impedance to determine cell shape after deformation. Differences between normal RBCs and stiffer spherical RBCs (treated with SDS and Glutaraldehyde) were identified. Owing to variations in the electrical impedance signal with the position in the channel, off-centre particles were discarded (based on velocity), corresponding to around 50% of total events.

In this paper we describe a high throughput single-cell shear flow deformability cytometer (sDC) that simultaneously measures the mechanical and electrical properties of single cells at a throughput of >100 s^-1^. The method does not use a separate sheath flow or high-speed cameras with associated data processing overheads. Cells are suspended in a viscoelastic buffer and pumped through a narrow channel, producing a shear force that induces cell deformation whilst also focusing cells into the channel centre^48^. Cell deformability and electrical properties are measured using integrated planar microelectrodes, at two discrete frequencies to give cell volume, shape and cell electrical properties. As a cell flows through a channel the electrical impedance is measured along two orthogonal axes to determine any shape change in the cell as it deforms (from sphere to ellipse) along with the electrical volume and opacity.

We demonstrate the utility of this technique by measuring the combined mechanical and electrical properties of HL60 cells under several different experimental conditions, including osmotic stress, Glutaraldehyde (GA) cross-linking, and cytoskeleton disruption. This new electro-mechanical phenotyping is simple and inexpensive. It does not require complex fluidics or sheath flow focusing, and demonstrates excellent correlation with sDC optical deformability measurements.

## Principle of operation

**Fig. 1:**
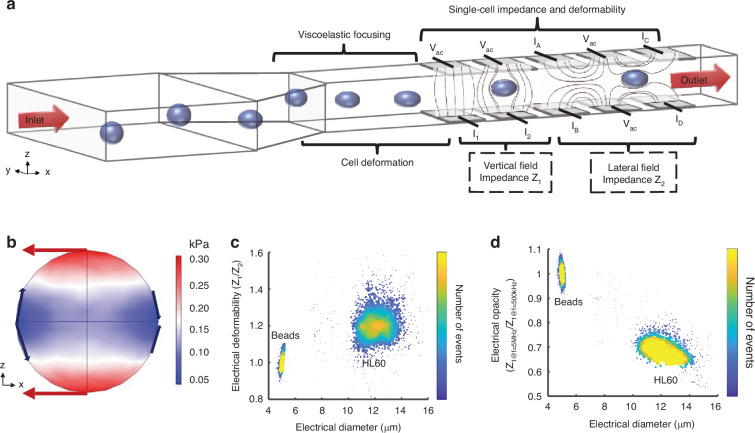
Principle of the single-cell electro-mechanical shear flow deformability cytometer. **a** Principle of the electro-mechanical cytometer. Cells suspended in a viscoelastic buffer flow along a microfluidic channel within which there are two sets of microelectrodes. One set measures the cell volume (Z_1_) whilst the second set measures cell deformation along the direction of flow (Z_2_). Cells are focused into the centre of the channel by the viscoelastic suspending fluid that is used to create the shear stress. **b** shows the shear stress on an undeformed sphere in a viscoelastic fluid (0.5% w/v methyl cellulose, 0.015 Pa·s, density 1005 kg/m^3^, flow rate 10 µl/min, particle radius 6 µm), see ESI for further details. Arrows indicate direction of the local force. Density plots of electrical deformability (**c**) and electrical opacity (**d**) as a function of electrical diameter for HL60 cells (*n* = 2000) at a flow rate of 10 μl/min. 5 µm rigid beads included as reference particles in both cases

The working principle of the system is shown in Fig. 1(a). Cells are suspended in a viscoelastic fluid (0.5% w/v methylcellulose in Dulbecco’s Phosphate Buffered Saline DPBS) and pumped through a micro-channel (40 μm wide, 28 μm high) at a flow rate of around 10μl/min. The viscoelastic fluid exerts a shear stress on cells as they enter the channel, leading to high deformation at low flow rates (Fig. 1b) and (Supplementary Fig. 1). It also partly focuses the cells into the centre of the channel, minimising the positional dependence of the impedance signals, eliminating particle-particle and particle-channel contact, and improving the stability of the cells in the flow. The change in cell shape is determined from the impedance signal recorded from the two sets of electrodes, as shown in the figure. One set generates an electric field orthogonal to the flow, while the second set creates a field along the flow direction as shown by the vertical and horizontal electric field lines in Fig. 1(a). Cells first enter the vertical field region (electrode currents I_1_ and I_2_) where the cell volume is determined. The second set of electrodes generates a field along the flow direction (I_A_, I_B_ and I_C_, I_D_) measuring the particle cross-section along the flow direction from which the deformation of the cell is determined.

At low AC frequencies (<500 kHz in saline) cells behave as electrical insulators so that the impedance signal is proportional to the electrical volume. An absolute volume measurement is obtained by scaling the impedance signals using solid polystyrene reference particles of known volume, mechanical and electrical properties^45^. Cell deformability is defined as the ratio of the low frequency vertical to lateral impedance (**Z**_**1**_**/Z**_**2**_) (Fig. 1a). An example scatter plot of electrical deformability vs diameter for HL60 cells is shown in Fig. 1c. The electrical deformability of the undeformed calibration beads is set to 1.0; softer cells have values greater than 1.0.

The impedance at higher frequencies (5 MHz) provides information on the electrical properties of the membrane and cytoplasm. The ratio of this impedance to the low frequency impedance is termed the electrical opacity and normalises for cell volume. Figure 1d shows a scatter plot of electrical opacity vs electrical diameter for the HL60 cells. The data is scaled to the opacity of homogenous solid dielectric beads (equal to 1.0 by definition)^45^. Biological cells with membranes have values lower than 1.0, and changes in opacity correlate with membrane capacitance and cytoplasmic properties.

## Simulation

**Fig. 2:**
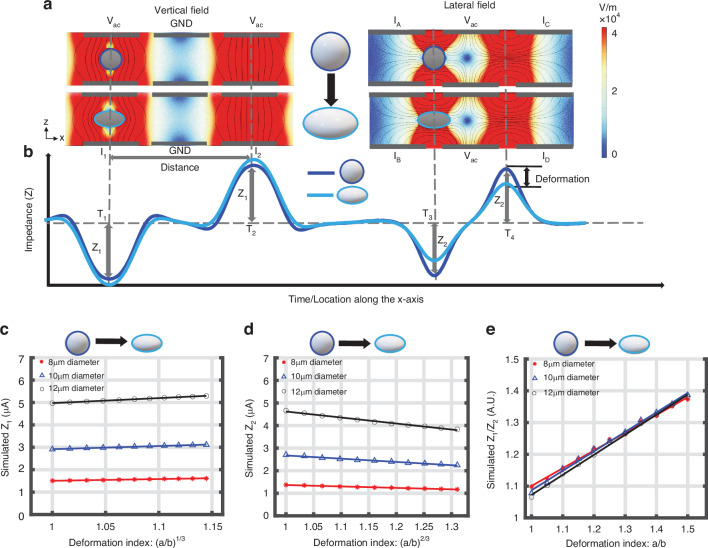
Simulation of electrical impedance and cell deformation. **a** Side view (z-x) of the vertical and lateral electric field for an applied voltage of ±1 V. with a solid dielectric sphere and ellipsoid. Colour scale is the magnitude of the electric field (V/m). **b** The two sets of electrodes create an impedance signal consisting of two sets of antisymmetric bimodal Gaussians. **c** Sensitivity plots for vertical impedance Z_1_ and (**d**) lateral impedance Z_2_ as a function of the deformation index (major/minor axis) for three different sizes of particles. **e** Electrical deformability (Z_1_/Z_2_) as a function of the deformation index for three different sizes of particles. Particles were modelled as homogenous solid dielectric objects with specific conductivity and permittivity (ε_p_ = 2.5ε_0_ and σ_p_ = 2.7 × 10^−3 ^S/m) and a frequency of 500 kHz. Simulated channel dimensions are 200μm long, 28 μm high and 40 μm wide

In order to understand the relationship between electrical impedance and cell deformation, a series of finite element simulations of the system were performed. The numerically calculated electric field for the electrodes is shown in Fig. 2a along with the resulting time-dependent impedance signals, Fig. 2b. The figure shows that in the first set of electrodes (Z_1_) the electric field is orthogonal to the flow and the impedance of an ellipsoid is slightly larger than the undeformed sphere. Z_2_ measures the cross-section of the particle as it flows along the channel. For an ellipsoid, less current is blocked compared to a sphere resulting in a reduction in Z_2_. The impedance of an elongated object is therefore different from that of an undeformed object. Finally, the electrical deformability is determined from the ratio of the two impedances, Z_1_/Z_2_.

The impedance of a particle depends on its vertical position in the channel^49^ and several different approaches have been adopted to correct/compensate for this^50–52^. On our devices this error is minimised through the use of an optimised electrode arrangement (see Supplementary Fig. 2a). The viscoelastic medium ensures that cells are focused into the central region of the channel as evidenced by the particle velocity data and particle tracking images shown in Supplementary Fig. 3. This means that the impedance from the first set of electrodes (Z_1_) provides a good estimate of cell volume. The lateral impedance (Z_2_) is more sensitive to both position and cell deformation (see Supplementary Fig. 2b and c), but the viscoelastic focusing again ensures that most particles are confined to the central region of the channel. The vertical impedance Z_1_ is also used to measure the electrical properties of the cells because these electrodes minimise the positional dependence of the signal.

In order to quantify the expected change in impedance as particles deform, the current in the channel was numerically calculated for a series of solid ellipsoids with different major/minor axes. In each case the particle volume was kept constant. Defining major axis *a* and minor axes *b* = *c*, the ratio between *a* and *b* (= *c*) was varied from 1.0 (sphere) to 1.5 (ellipse) and the electrical current calculated. This allowed the deformation index, defined as the ratio of major axis to minor axis (*a*/*b*) to be calculated.

For a fixed volume object the cross-sectional area of the vertical projection (A_v_) as measured by *Z*_*1*_ is given by Eq. (1), whilst the cross-sectional area of the horizontal projection (A_h_) is given by Eq. (2) (for derivation see Supplementary).

1 $${A}_{v}=\pi {\left(\frac{3* {vol}}{4* \pi }\right)}^{\frac{2}{3}}* {\left(\frac{a}{b}\right)}^{\frac{1}{3}}$$

2 $${A}_{h}=\pi {\left({\left(\frac{3* {vol}}{4* \pi }\right)}^{\frac{1}{3}}\right)}^{2}* {\left(\frac{b}{a}\right)}^{\frac{2}{3}}$$

In other words, for a fixed cell volume, A_V_ scales with (*a/b*)^1/3^ and A_H_ scales with (*b/a*)^2/3^

Figure 2c shows the vertical impedance Z_1_ as a function of the cube root of the deformation index (*a*/*b*) for different particle diameters (Eq. (1)), demonstrating that the overall change is small, particularly for smaller particles. The trend is an increase in impedance with aspect ratio. To allow direct comparison, Fig. 2d is a plot of Z_2_ against (*a/b*)^(2/3)^, i.e. the horizontally projected area (inverse of Eq. (2)). This shows that the horizontal impedance (Z_2_) decreases as the aspect ratio increases. Importantly particle deformability determined from the ratio of the two impedances (Z_1_/Z_2_) is almost independent of particle size (see Supplementary for derivation) and is linearly proportional to particle aspect ratio (*a*/*b*), as shown in Fig. 2e.

To summarise, a shear flow electro-mechanical cytometer with a specific electrode configuration can be used to measure the shape of a cell as it deforms in a viscoelastic flow along a channel. Simultaneously the electrical impedance of the cell is measured at two probe frequencies to extract cell volume and cell electrical opacity. In order to evaluate the devices a set of measurements of the properties of HL60 mammalian cells were performed, including exposing cells to osmotic shock, cross-linking of the membrane and disruption of the cell cytoskeleton.

## Experimental

### Osmotic shock

**Fig. 3:**
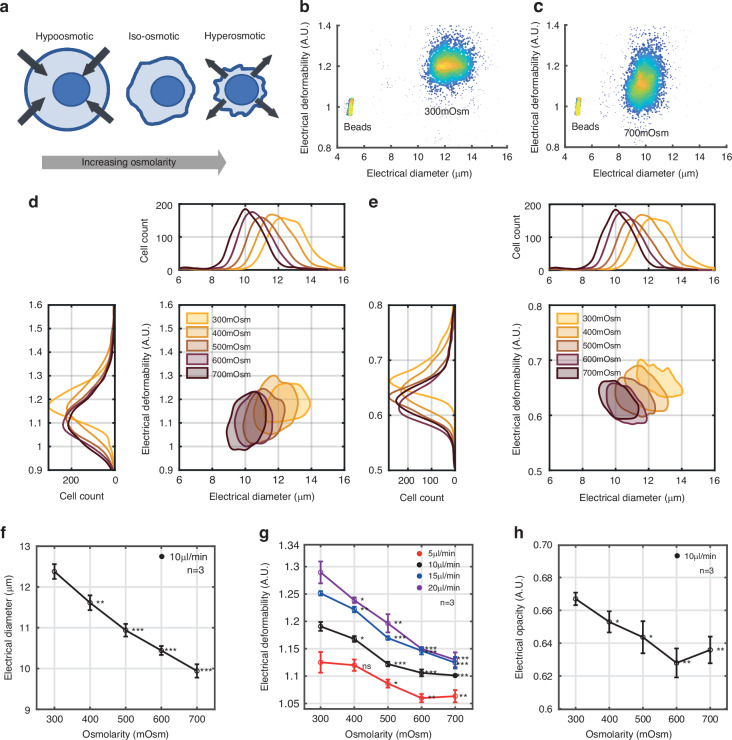
Effect of osmotic pressure on cell deformability and opacity. **a** Increasing the osmotic pressure causes cells to shrink leading to an increase in membrane folds. **b** and **c** example density scatter plots of electrical deformability vs electrical diameter for 300 mOsm and 700 mOsm buffers at a flow rate of 10 μl/min. **d** Contour plots (50% density) of electrical deformability vs electrical diameter for different osmolarities (flow rate 10 µl/min). **e** electrical opacity vs. electrical diameter for different osmolarities (flow rate 10 µl/min). Data is representative of one experiment. **f** Mean electrical diameter as a function of osmolarity (*n* = 3). **g** Mean electrical deformability as a function of osmolarity for different flow rates (*n* = 3). **h** Electrical opacity vs osmolarity at a flow rate of 10 µl/min, (*n* = 3). P-values (student’s t-test) are relative to cells measured at isotonic osmolarity (300 mOsm). ***p < 0.001, **p < 0.01, *p < 0.05, and ns not-significant. Number of cells in each group is approximately 2000

Mammalian cells exposed to hyperosmotic solutions (higher than 300 mOsm) rapidly shrink and become much stiffer as the volume of the cytoplasm is reduced, leaving the majority of the internal volume occupied by the cell nucleus^53^, as shown in Fig. 3a. The increased intracellular molecular crowding leads to an increase in cell stiffness and an increase in membrane folds which would manifest as an increase in the cell membrane capacitance. Therefore, a series of osmotic shock experiments were performed on HL60 cells using buffers of varying osmotic strength but with constant electrical conductivity. Prior to measurement, each group of cells was exposed to a different osmolarity-adjusted methyl cellulose (MC) buffer for 10 minutes.

Figure 3 shows representative scatter plots of electrical deformability at 300 mOsm (b) and 700 mOsm (c) at a flow rate of 10 µl/min. Contour plots (50% density) of deformability *vs* electrical diameter for different osmolarities is shown in Fig. 3d demonstrating that both the electrical diameter and deformability reduce with increasing osmolarity. Figure 3e shows contour plots of electrical opacity vs. diameter for different osmolarity showing a decreasing trend in opacity with increasing osmolarity. This is consistent with the expected increase in membrane surface folds, which in turn leads to an increase in cell membrane capacitance (i.e. the opacity at 5 MHz decreases). Figure 3f summarises data for electrical diameter as a function of osmolarity at a flow rate of 10 µl/min (*n* = 3), where the diameter decreases with osmotic pressure, a trend that is independent of the flow rate (Supplementary Table 1).

Figure 3g shows how electrical deformability decreases with osmolarity at different flow rates (*n* = 3), demonstrating that the relative change increases with the flow rate (Supplementary Table 1) as the higher shear stress increases the deformation of cells. Finally, Fig. 3h summarises the electrical opacity changes with osmolarity (at 10 µl/min), demonstrating an increase in the membrane capacitance as the cells shrink. Different flow rates had little effect on the relative change in opacity. Both the deformability and size of HL60 cells decreased with increasing osmolarity consistent with previous reports^35^.

These results are consistent with Sukhorukov et al.^54^ who demonstrated that the membrane surface area reduces as cells expand in hypoosmotic solutions, with a reduction in cell membrane capacitance before reaching a limiting value. As the cells swell in hypotonic solution, microvilli disappear to compensate for increasing membrane area. By contrast exposure to hypertonic solutions leads to shrinkage of cells and collapse of the apical membrane onto the cortex^55^, so that the cells become much stiffer with an increase in cell membrane capacitance, as observed experimentally.

### Cross-linking

**Fig. 4:**
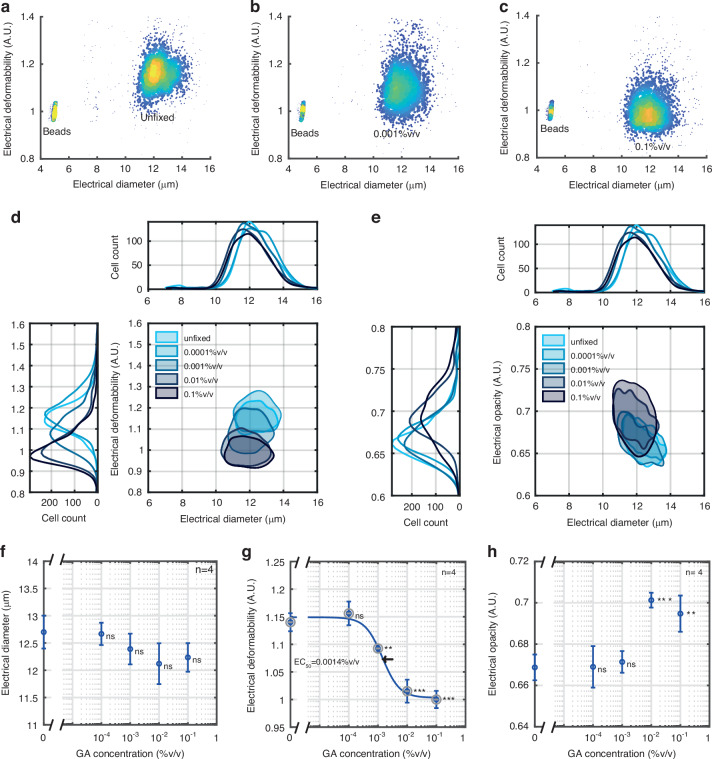
Effect of glutaraldehyde on cell deformability and opacity. Example density plots of electrical deformability as a function of the electrical diameter for (**a**) unfixed, (**b**) 0.001% v/v and (**c**) 0.1% v/v GA fixed HL60 cells at a flow rate of 5 μl/min. Contour plots (50% density) of electrical deformability (**d**) and electrical opacity (**e**) vs electrical diameter. Dose-response data for electrical diameter (**f**), electrical deformability (**g**) and electrical opacity (**h**) at 5 μl/min. Error bars are standard errors of the mean for *n* = 4. P-values (Student’s t-test) are relative to unfixed cells (0%v/v). EC_50_ for electrical deformability = 0.0014% v/v. The number of cells in each group was approximately 2000

It is known that cells become much stiffer after exposure to protein cross-linking using agents such as glutaraldehyde (GA) where deformability gradually decreases with increasing concentration^56,57^. To study the dose-response of GA, HL60 cells were treated with different concentrations and the stiffness compared with unfixed control cells. Cross-linking with GA significantly altered both cell deformability and cell opacity. Figure 4a, b and c show example scatter plots of electrical deformability vs electrical diameter at different concentrations of GA. At the highest concentration (0.1% v/v), the electrical deformability is around 1.0, similar to the control beads. Cell diameter was not influenced by GA treatment. Figure 4d shows contour plots (50% density) of electrical deformability vs diameter for different GA concentrations whilst Fig. 4e shows a similar plot for the electrical opacity. Dose-response curves are shown in (f)), (g) and (h). No change was observed in cell size but significant differences were observed in cell deformability even at small concentrations of GA. Fitting this data to a four-parameter Hill equation, gave a half maximum concentration, EC_50_ = 0.0014% v/v GA, consistent with deformability measurement made in spiral microchannels^56^. Increasing the flow rate to 10 μl/min, had a minimal influence on the electrical deformation, which remained similar to that at 5 μl/min (Supplementary Fig. 4a), with a similar EC_50_ value (Supplementary Fig. 4b).

Figure 4(h) summarises the mean electrical opacity vs GA (*n* = 4), where only the two highest concentrations (0.01% and 0.1% v/v GA) lead to significant changes in cell electrical opacity. It has been shown that treatment of red blood cells with glutaraldehyde leads to cross-linking of the protein networks that form the cell membrane and the cytoskeleton leading to an increase in opacity^58^. This increased opacity was linked to decreased cytoplasm conductivity and decreased membrane capacitance, both resulting from protein cross-linking consistent with Gagnon et al.^59^ who showed that GA cross-linking reduces membrane permittivity (from 10.5 to 3.8) for RBCs and Pribush et al.^60^ who showed that GA reduces the capacitance of RBC membranes.

### Cytoskeleton disruption

**Fig. 5:**
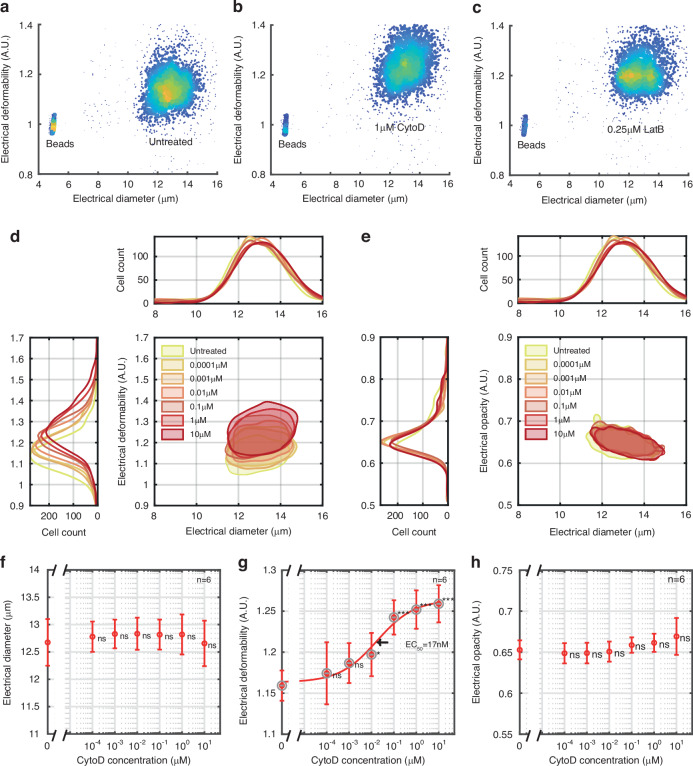
Effect of cytoskeleton disruption on cell deformability and opacity. Density plots of electrical deformability vs electrical diameter for (**a**) untreated, (**b**) 1 μM CytoD and (**c**) 0.25 μM LatB treated cells. Contour plots (50% density) of (**d**) electrical deformability (**d**) and electrical opacity vs electrical diameter (**e**) for CytoD-treated cells. Dose-response graph for electrical diameter (**f**), electrical deformability (**g**) and opacity (**h**). CytoD deformability EC_50_ for = 17 nM. Error bars are standard errors of the mean value of *n* = 6. P-values (Student’s t-test) are relative to untreated cells. All data collected at a flow rate of 5 μl/min

The mechanical properties of the cytoskeleton of cells were altered by treatment with Cytochalasin D (CytoD) or Latrunculin B (LatB). CytoD inhibits actin filament elongation by binding to the barbed ends of the filaments preventing polymerisation, resulting in loss of cytoskeletal structure and decreased stiffness. LatB modulates cell stiffness by binding actin monomers, preventing them from polymerising the actin filaments. Example scatter plots illustrating the effect of these compounds are shown in Fig. 5a, b and c for control, 1 μM CytoD and 0.25 μM LatB demonstrating that exposure leads to a decrease in cell stiffness, consistent with observations from other groups^35,45,61^. Contour plots of electrical deformability vs electrical diameter for different CytoD concentrations (at 5 µl/min) are shown in Fig. 5d, (Supplementary Fig. 5a for 10 µl/min). Cell diameter was not significantly affected by either Cyto D (Fig. 5f), or Lat B consistent with other reports^35^ although Guzniczak^56^ noted a small (10%) reduction in cell size measured by flow cytometry (FSC) for Cyto D as low as 10 nM. Dose-response curves were extracted from measurements of CytoD cells at different concentrations, yielding half-maximal concentrations (EC_50_) of 17 nM (@ (5 µl/min), Fig. 5g or 11 nM (@ 10 µl/min) in good agreement with the previously reported values of 13.5 nM^53^. Figure 5e shows that electrical opacity is unaltered by exposure to CytoD, implying that destabilisation of the cytoskeleton has no statistically observable effect on the membrane or cytoplasmic properties as determined from the electrical opacity. This contrast with the results of Jaffe and Voldman^38^ who measured the electrical properties of 262 cells exposed to Cyto D using a dielectrophoresis spring system. They observed differences between control and treated cells although no absolute values of the electrical properties were reported. These data were obtained using multiple frequencies up to 25 MHz and furthermore, their system does not expose cells to shear stress making direct comparison difficult.

### Correlating optical and electrical deformability

**Fig. 6:**
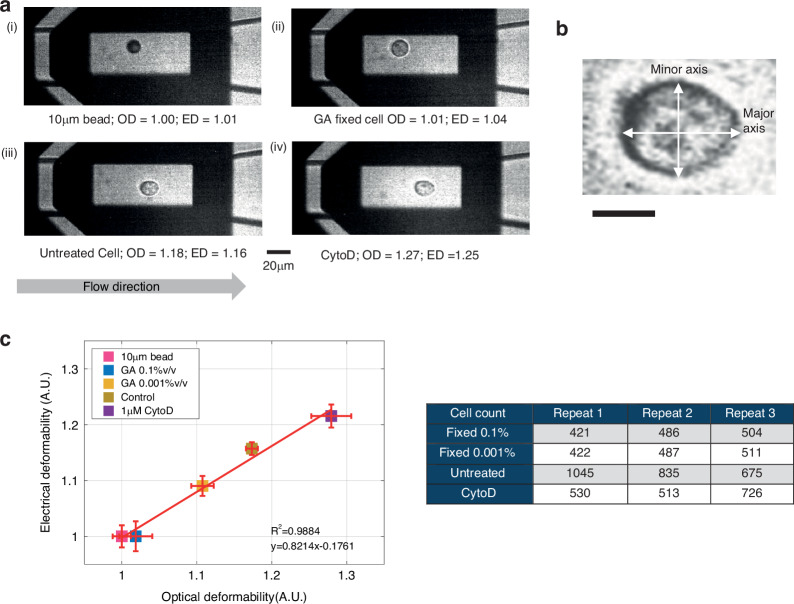
Correlation between electrical and optical deformability. **a** Optical images of particles and cells at the end of a channel at a flow rate of 10 μl/min. Images were recorded with a camera with cell contours and deformability extracted from the images off-line. Optical Deformability (OD) of the individual particles are shown (i) Solid particle (ii) GA fixed HL60 cells (iii) Control (untreated) cell (iv) CytoD treated cell. **b** Example image of a single HL60 cell deforming in the channel at a flow rate of 10μl/min; scale bar = 10 µm. **c** Correlation between the Electrical Deformability (ED) and Optical Deformability (OD) for HL60 cells (Control, GA and CytoD treated) together with 10 μm solid particles. Data is the mean of 3 biological repeats (*n* = 3). The correlation between the two methods is given by the solid line (R^2^ = 0.99)

Having demonstrated that the system is able to discriminate between cells before and after treatment with different compounds that affect cell stiffness, the question of correlation with the more conventional optical method remains. To address this, the system was modified to include an imaging capability consisting of a high-speed camera and an LED light source triggered by the impedance signal (see Fig. 6 supplementary). An example image of cells flowing along the channel captured using this method is shown in Fig. 6. Note that the throughput in this case is much lower than for the electrical deformability method due to the limitations of our optical system. Single-cell images were post-processed and referenced to the solid calibration beads to obtain the optical deformability (OD). The images demonstrate that the cells deform in the flow and that this deformation depends on the chemical treatment. The correlation between the two methods was evaluated by plotting Electrical Deformability (ED) against Optical Deformability (OD). Figure 6(c) shows this plot for untreated cells, GA fixed cells and CytoD-treated cells. Of note is that the solid polystyrene beads are perfectly spherical and have a deformability of 1.0. However, GA fixed cells are not perfect spheres and therefore when imaged have an optical deformability very slightly greater than 1.0. Electrically their deformability is measured in a different way and is close to 1.0. In this plot, each data point is the mean of several hundred cells (see Table in the figure), repeated three times (*n* = 3). As shown by the plot, the correlation between the ED and OD is excellent.

## Summary and Conclusion

This paper describes a new shear flow deformability cytometer (sDC) that measures the electro-mechanical properties of single cells at high throughput. Unlike many devices used to characterise cell electrical and mechanical properties, it does not rely on a cell squeezing through a narrow pore. The device can characterise cells at up to 100 per second, similar to the sDC systems reported elsewhere. The cytometer was used to characterise changes in cells exposed to different chemical/physical stress demonstrating concordance with other optical techniques. The correlation between this electrical method and optical deformability based on 2D imaging is excellent. Changes in cell properties due to osmotic swelling were clearly measurable although the sensitivity of the device is lower than reported by others^30^ which could be due to the incomplete focusing of cells in the viscoelastic fluid, where the introduction of a sheath flow into the device is likely to lead to significant improvements.

Cross linking of the cell membrane with GA clearly makes the cells significantly stiffer but also modifies the cell opacity. This may reflect changes in cell membrane properties and/or cytoplasmic properties and a fuller understanding of this would require the use of broad band frequency analysis of single cells followed by fitting to the multi shell model^45^. The system demonstrates that cytoskeletal disrupting compounds markedly change cell stiffness but that changes in cell electrical properties are not statistically significant, as determined within the constraints of the two frequencies used here. The EC_50_ for Cyto D corresponds well to published data (around 15 nM). This is significantly lower than the minimum amount reported for cell transiting through a constriction channel where the mechanical properties of cells treated with Cyto D were only statistically significant above concentrations of 1 μM^30^.

This new cytometer has several advantages. The technology is relatively simple and does not require high-speed cameras. Detection of single-cell events by impedance could be used to trigger image capture using a simple low-cost CMOS camera. The system could be useful for drug screening or even as a test to diagnose disease that affect the electro-mechanical properties of cells. Furthermore, high-speed signal processing of the electrical signals could lead to the development of a high-speed cell sorting systems furthering our understanding of cell heterogeneity and the links between electro-mechanical phenotype and disease at the single cell level.

## Methods

### Impedance analysis system

A diagram of the system is shown in Supplementary Fig. 6. A glass impedance chip containing sets of microelectrodes is mounted in a custom PCB that contain the drive and sense electronics. As shown in the diagram and by the photograph of the chip, it has multiple electrodes to measure the electrical properties of the cells. It also has an optical window (34 μm wide) after the electrode region which is used to image the cells. The electrical properties of cells were measured using two superimposed frequencies 500 kHz (lf) and 5 MHz (hf). The current change from the electrodes is converted to voltage with a custom trans-impedance amplifier. A lock-in amplifier (HF2LI, Zurich Instruments) demodulates the signals separating the real and imaginary parts. Signals from each cell are processed and analysed using custom programs written in MATLAB. Prior to each measurement the chip was flushed with 1 M sodium hydroxide for 10 minutes to remove any residue followed by rinsing with deionised water. All buffers were filtered through a 0.22 µm filter to avoid blockage by larger particles. The sample suspension was diluted to ensure that on average only one particle passed through the detection volume (360 µm × 40 µm × 28 µm), with a typical cell concentration of around 500 cells/ µl, mixed with beads at 200 beads/µl. Samples were measured at a flow rate of between 5 and 20 µl/min, with a throughput of around 200 particles per second. Higher flow rates could be used but the signal quality is reduced due to quantization errors because of the limited sample rate on the impedance lock-in amplifiers.

### Impedance chips

A photograph of the impedance chip (20 mm ×15 mm) is shown in Supplementary Fig. 6. The chip is made from two glass wafers with nine Pt electrodes patterned on each side. One wafer is patterned with a thick resist to create a channel and the wafer pair bonded and diced to give individual chips. Fluid inlet and outlet holes are drilled using a laser. The measurement channel is 360 µm long, 40 µm wide and 28 ± 2 µm high. All electrodes have a width of 30 µm with a 10 µm gap.

### Image processing

Images of cells were captured using a high-speed camera and post-processed in MATLAB. First the cell outline was identified followed by the determination of the major axis and minor axis. The raw images were contrast-enhanced and converted into grayscale images. Edge detection was used to determine the cell perimeter, which was fitted to an ellipse by computing second-order moments using the function *regionprop* to give the major axis, minor axis and centroid position. The Optical Deformability (OD) of the cell is defined as the ratio of major axis to minor axis^43^, which is 1.0 for a spherical object.

### Cell culture

HL60 were cultured in RMPI 1640 + Glutamax with 10% Fetal Bovine Serum and 1% Penicillin-Streptomycin. Cell stock was kept in liquid nitrogen. Cells were thawed in a 37 °C water bath, washed and resuspended at a concentration of 5 × 10^5^ cells/ml in fresh 20% FBS medium. Cell concentration was kept between 10^5^ and 10^6^ cells/ml and cells were maintained in 10% FBS medium. Cells were collected every two days after seeding when they were in the exponential growth phase (cell concentration never exceeded 1000 cells per µl).

### Methyl cellulose (MC) buffer

Cells were resuspended in a buffer containing 0.5% w/v Methyl Cellulose (MC) in DPBS buffer made by dissolving dry MC powder (1 g in 200 ml) as follows: Heat 70 ml of DPBS to 80 °C, add 1 g of MC powder and stir gently to disperse the powder. Add 130 ml room temperature DPBS to the mixture with constant stirring to avoid clumping or aggregation (without heat). The MC mixture begins to hydrate as the temperature decreases and becomes jelly like. After the solution has cooled to room temperature, place the mixture in the fridge for two hours to fully hydrate the MC. Buffers were stored in the refrigerator and were brought to room temperature before use, and filtered through a 0.22 μm filter.

### Glutaraldehyde (GA)

For GA experiments, the cell concentration was around 500 cells/µl. Cells were first suspended in PBS with different concentrations of Glutaraldehyde for 30 minutes at room temperature. After incubation, cells were centrifuged and resuspended in 0.5% w/v MC in DPBS for deformability measurements. The same impedance chip was used for all GA experiments.

### Osmotic shock

The osmolarity of the buffers was measured with a micro-osmometer (Loser). The osmolarity was increased by the addition of different amounts of D-mannitol to give 400, 500, 600 and 700 mOsm solutions. The conductivity of the solutions was measured using a conductivity meter. HL60 cells at a density of around 500 cells/µl were centrifuged at 180 g for 5 min and then resuspended in the different buffers for 10 mins at ${37}^{{\rm{o}}}{\rm{C}}$ (in an incubator). The same impedance chip was used for all osmotic shock experiments. Biological repeats were carried out with the same stock solutions to maintain an identical osmolarity for each group.

### CytoD

Cells (1 ml volume @ 500 cells/μl) were centrifuged and resuspended in 0.5% w/v MC buffer. CytoD solutions were made by dissolving the dry powder in dimethyl sulfoxide (DMSO) to give solutions of different molarity (2000, 200, 20, 2, 0.2 and 0.02 μM) each with the same amount of DMSO (0.5% v/v). Cells were mixed with 5 μl of these stock solutions. Untreated cells (control) were also exposed to 0.5% v/v DMSO. Cells were exposed to CytoD at 37°C in an incubator for 10 minutes. CytoD is reversible therefore cells were not washed before measurement. Dose-response curves were obtained at flow rates of either 5 or 10 μl/min. Biological repeats were made on different days and within ten cell passages. The same impedance chip was used for all experiments, and the CytoD solutions were freshly prepared each time.

### LatB

Stock solutions were made by dissolving 1 mg of Latrunculin B dry powder in 1 ml DMSO. Cells were centrifuged and resuspended in 0.5% w/v MC buffer. LatB concentrations was 0.25 μM. Cells were exposed to LatB for 30 minutes, at 37°C in an incubator and were not washed prior to measurement. Biological repeats were conducted on different days and within ten cell passages.

## Supplementary information

Supplementary figures and material

## Data Availability

Data for this publication are obtainable from 10.5258/SOTON/D3151.
