# Supplementary material for: Single-cell electro-mechanical shear flow deformability cytometry
Source: Microsyst Nanoeng. 2024 Nov 22;10:173. doi: 10.1038/s41378-024-00810-5 (PMC11582679; doi:10.1038/s41378-024-00810-5)
Supplement: Supplementary file 1 — Supplementary figures and material [file 41378_2024_810_MOESM1_ESM.docx]

**Supplementary**


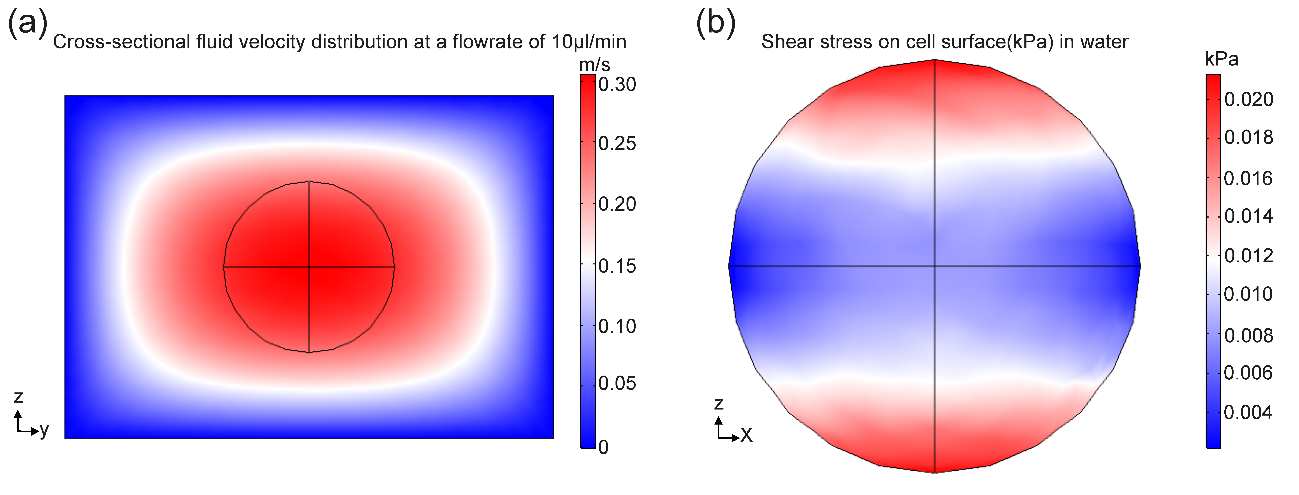


**Supplementary Figure 1** (a) Velocity profile of the fluid in the channel at 10µl/min. The cross section is 30µm high (z-axis) and 40µm wide (y-axis). (b) Simulation of the shear stress on the surface of a particle suspended in a low viscosity fluid (water) of 1mPa·s and a density of 1000 kg/m^3^ at a flow rate of 10µl/min. The channel was 300μm long with a cross-section of 30×40μm. A 6μm radius spherical particle is suspended in the centre of the channel. Compared with water, the tangential stress on the particles in methylcellulose-PBS solution increases from 0.02kPa to 0.3kPa, sufficient to deform the cell (Figure 1b).


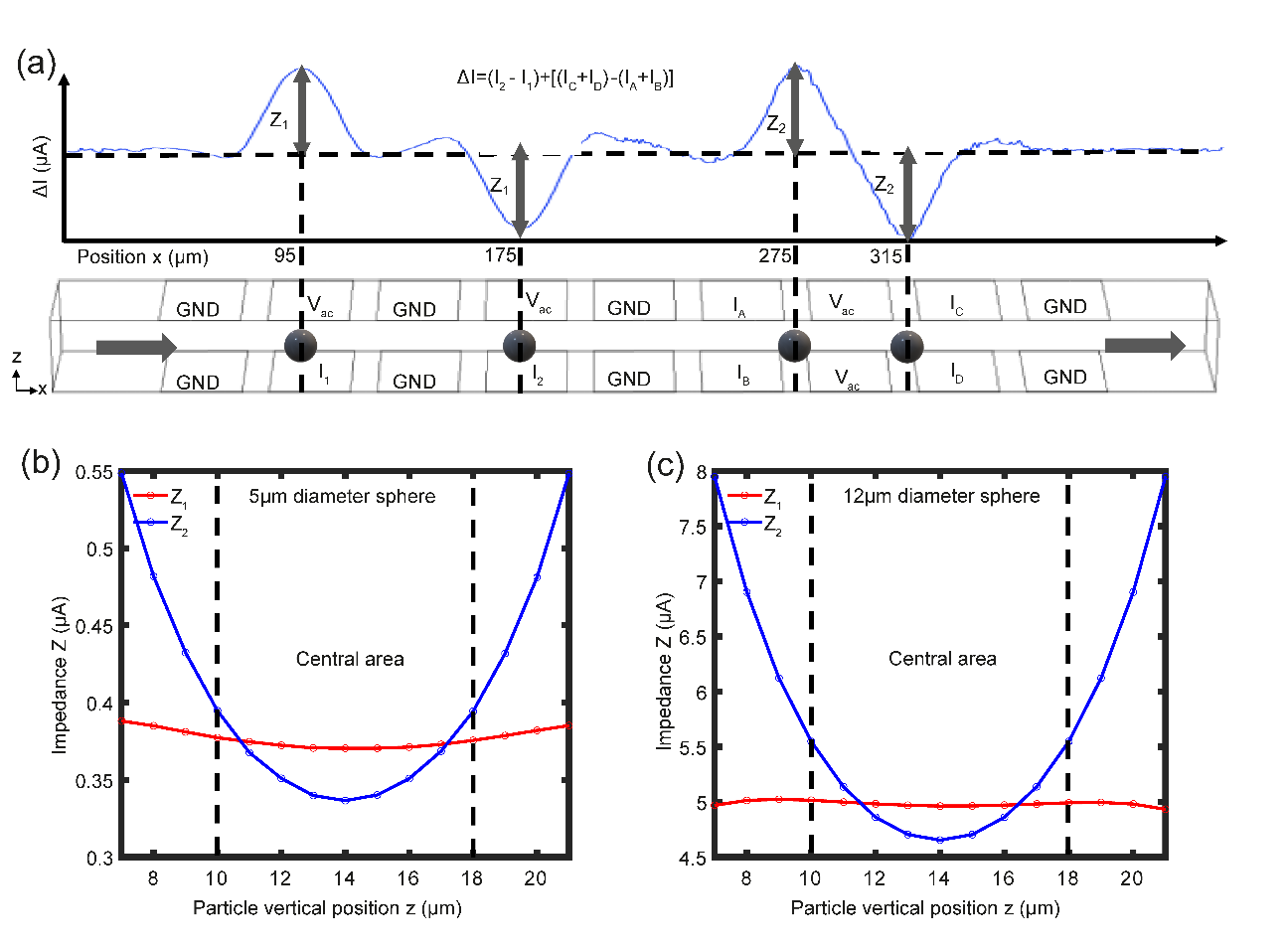


**Supplementary Figure 2** (a) Simulation of the differential current (arbitrary units) for a single solid homogeneous dielectric particle moving along the channel centre. The channel is 28um high and the electrodes are connected as shown. (b) Magnitude of the vertical impedance Z_1_ (red) and lateral impedance Z_2_ (blue) as a function of vertical position in the channel (z-axis) for a 5µm diameter particle. (c) as for (b) but for a 12µm diameter particle. Note that the z-axis range is from 7µm to 21µm. Given that the channel height is 28um, the 12µm particle will be in contact with the channel wall at the extremes which is unlikely in practice. The simulations show that Z_1_ is almost invariant with particle position unlike Z_2_.


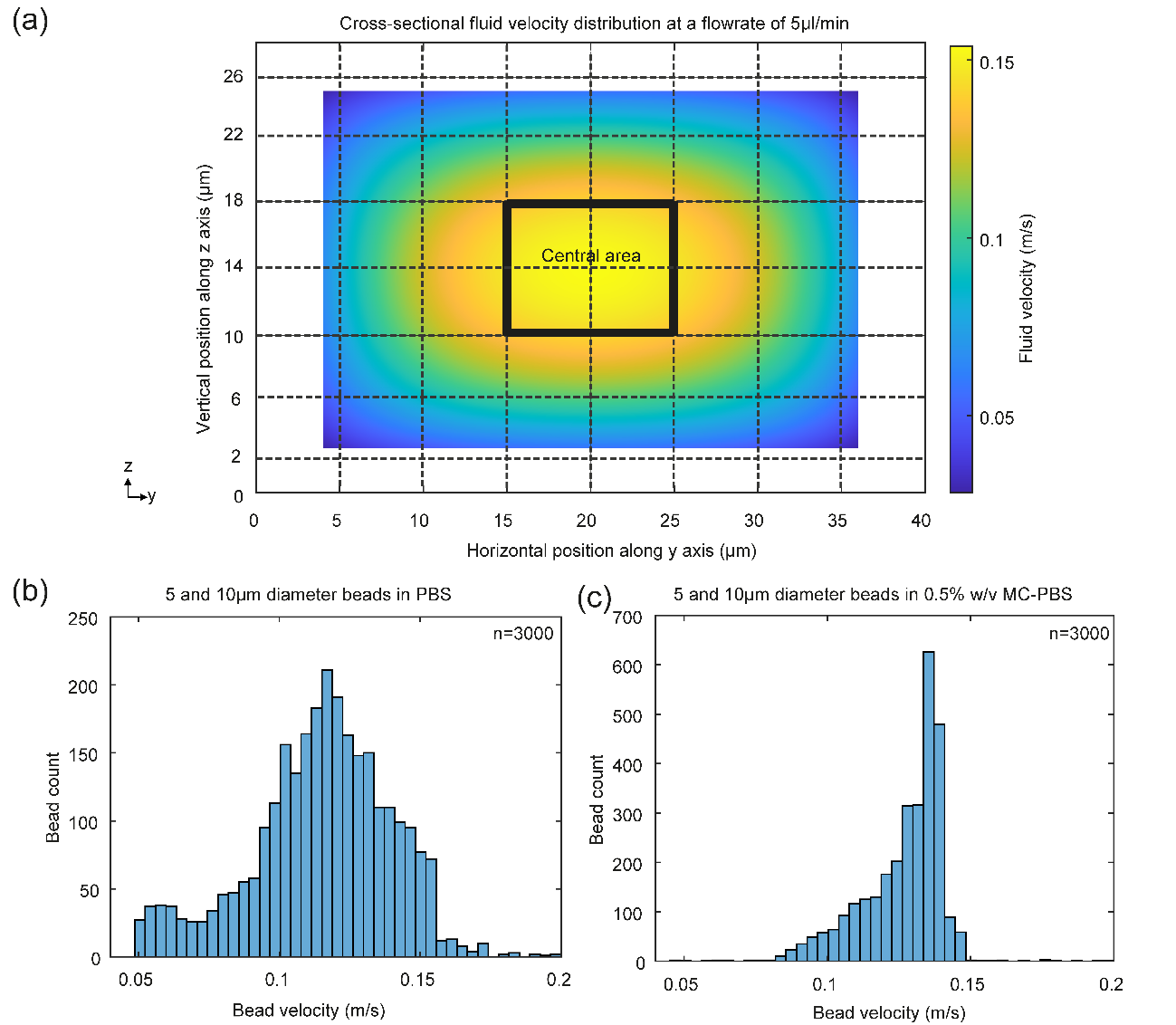


(d)


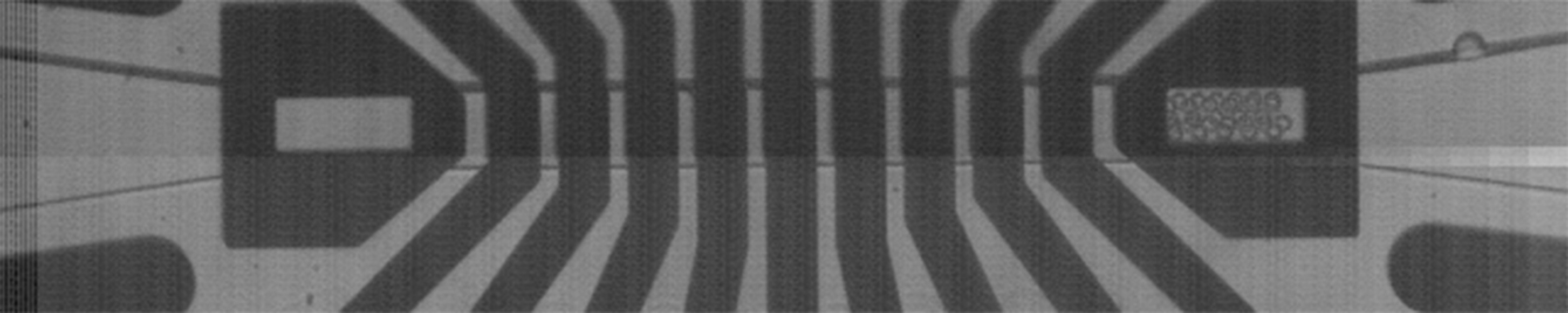

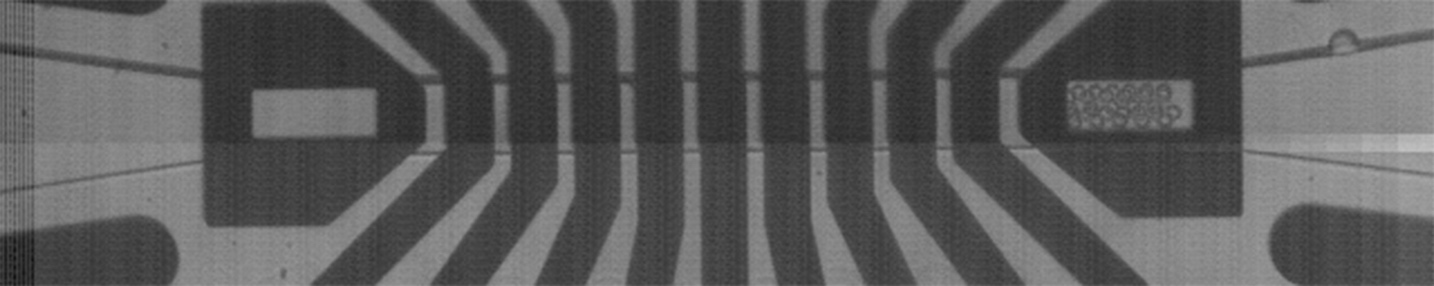


(i)


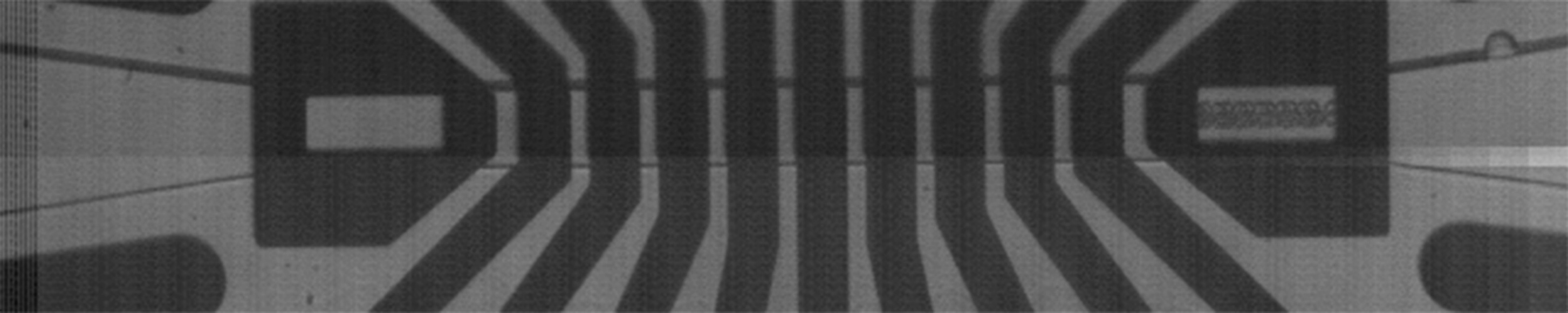

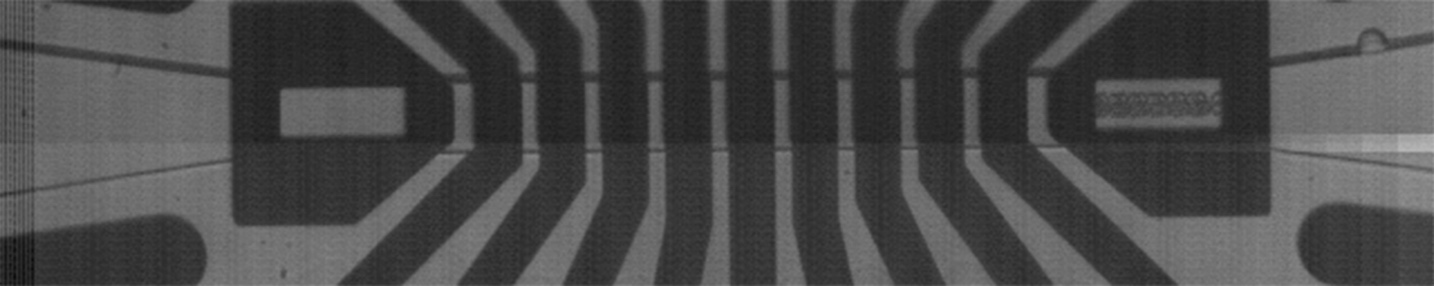


(ii)

**Supplementary Figure 3** (a) Numerically calculated fluid velocity profile for the 28µm×40µm cross-section channel at a volumetric flow rate of 5µl/min with saline of fixed viscosity. (b) Histogram showing the experimentally measured velocities of a mixture of 5 µm and 10µm diameter beads (suspended in PBS) at a volumetric flow rate of 5µl/min (approximately 3,000 particles); (c) Histogram for the same particles suspended in 0.5%v/v MC-PBS at a flow rate of 5µl/min (3,000 particles). The data shows that the MC-PBS buffer focuses the majority of the particles into the central region of the channel (square box in (a)). (d) Stacked high-speed images of 10μm diameter beads suspended in (i) PBS solution and (ii) MC (5μl/min) demonstrating focusing consistent with (b) and (c). Scale bar =30µm.


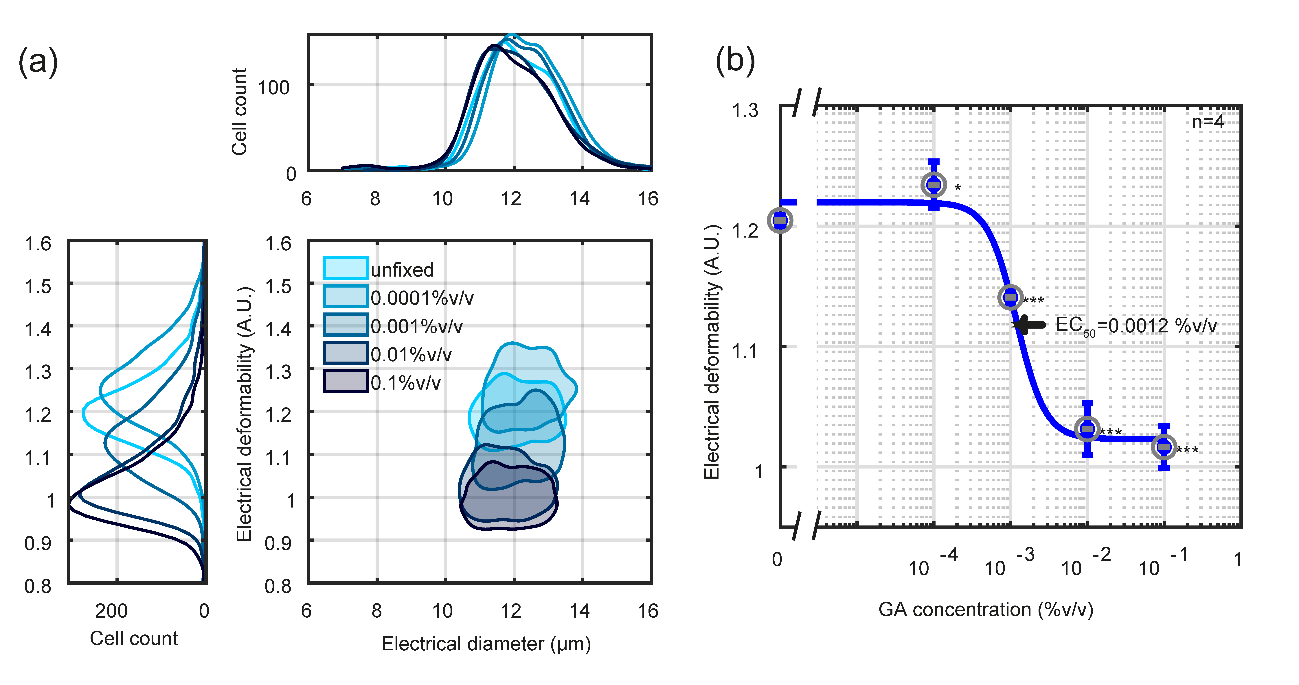


**Supplementary Figure 4** (a). Electrical deformability of GA fixed cell versus electrical diameter at a flow rate of 10μl/min. (b) Dose-response curve and Hill equation fit for electrical deformability at a flow rate of 10μl/min giving EC_50_ = 0.0012% v/v. Error bars are standard errors of the mean (*n*=4). P-values (Student’s t-test) are relative to the value for unfixed cells (0%v/v).


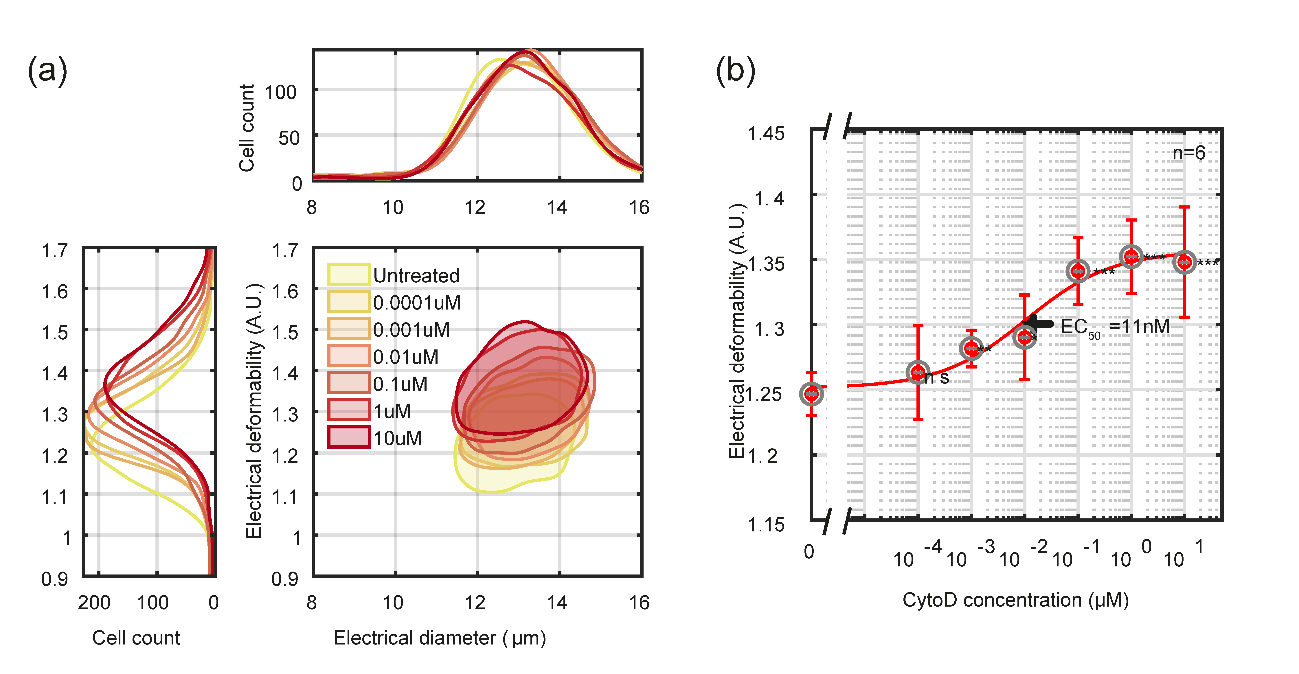


**Supplementary Figure 5** (a). Electrical deformability of CytoD treated HL60 cells at a flow rate of 10μl/min. (b) Dose-response plot of electrical deformability at a flow rate of 10μl/min giving EC_50_ = 11nM. Error bars are standard errors of the mean value of n=6. P-values (Student’s t-test) are relative to untreated cells.

**
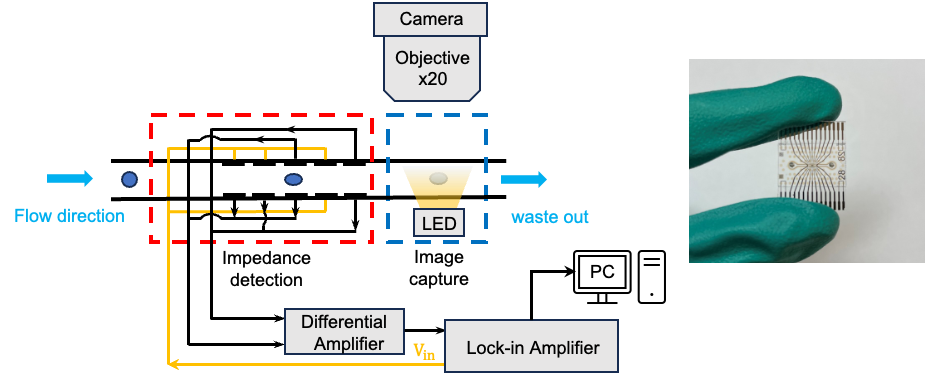
**


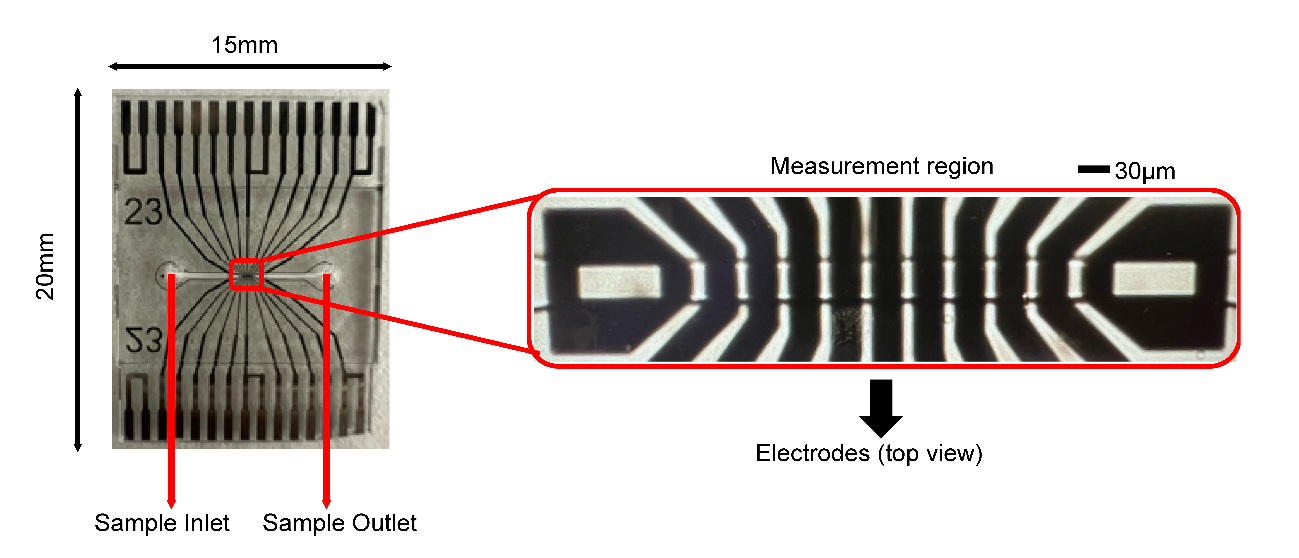


**Supplementary Figure 6.** Diagram of the experimental system. The cell mechanical and electrical properties are measured in the central region of the chip. The chip also has a window in the metal at each end through which the cell can be optically imaged.


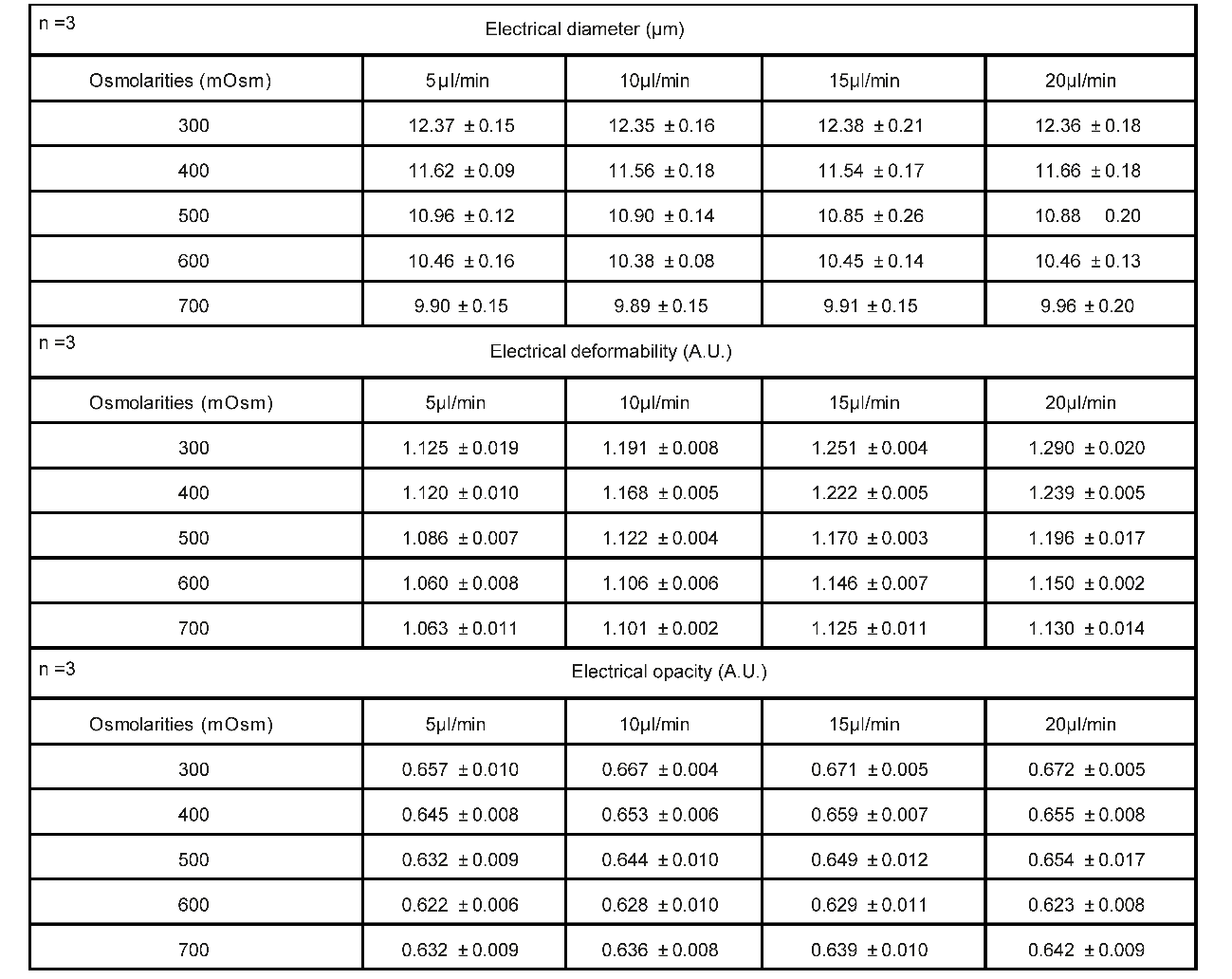


**Supplementary Table 1** Summary of the average electrical diameter, deformability and electrical opacity of cells in buffers of different osmolarity at increasing flow (*n*=3).

**Particle deformation calculations**

The forces deforming particles in a fluid can be decomposed into normal and tangential surface stresses. The drag force produces a normal stress $F_{d}$, which acts perpendicularly on a cell surface. At low Reynolds numbers, this can be estimated from Equation 1, where $v_{s}$ is the fluid velocity relative to the sphere (slip velocity), r is the radius of the particle, and $\eta$ the viscosity of the fluid.

$F_{d}\cong6\pi v_{s}\eta r$ (1)

The normal stress is highest at the rear and front of cells, but small on the surface close to the channel wall. This appears as a force in two directions, pushing a cell from the back and pulling the cell at the front, but is not the main contribution to cell elongation. Cells are mainly deformed by the tangential stress which is highest on the surfaces close to the channel walls. This stress is mainly composed of shear stress ($F_{s}$), which is generated by the velocity gradient and viscosity from the fluid in a narrow channel. The shear stress is given by Equation (2) where γ' is the shear rate, which is proportional to the fluid velocity ν.

$F_{s}\cong\gamma' \eta\left( 4\pi r^{2} \right)=2\pi\nu\eta r$ (2)

Increasing the viscosity of the suspension increases the shear stress. In addition, an increase in flow rate enhances the velocity gradient within the channel, thereby increasing shear stress.

The force on a spherical particle in a microfluidic channel was analysed by finite element simulation.

Supplementary Figure 3b shows the force on a spherical particles in a low-viscosity fluid (water). The tangential stress acting on the sphere surface is highest at the top and bottom of the sphere because the velocity gradient in the vertical direction (z-axis) for a shallow channel is higher than the transverse velocity gradient (y-axis direction). In addition, due to the no-slip condition between the sphere and the channel wall, the highest tangential stress occurs in the region closest to the channel wall. For water the peak tangential stress acting on the sphere surface is 0.02 kPa. Increasing the viscosity increases the tangential and normal stress on the surface of the particle. Figure 1b (main text) shows the force on the surface of a sphere for 0.5% w/v Methylcellulose-PBS solution (viscosity = 15mPa·s) at a flow rate of 10µl/min. The tangential stresses increase from 0.02kPa to 0.3kPa, sufficient to deform the cell.

**Derivation of equations (1) and (2)**

Consider an ellipsoid with axes: *a*, *b*, *c* and set *b = c* (prolate ellipsoid). Separate out *a/b* as the deformability index:

$Volume=\frac{4}{3}\pi abc=\frac{4}{3}\pi ab^{2}=\frac{4}{3}\pi\left( \frac{a}{b} \right)b^{3}$;

Rearrange to $b^{3}=\frac{3*vol}{4*\pi}*\frac{b}{a}$ and therefore $b=\left( \frac{3*vol}{4*\pi} \right)^{\frac{1}{3}}*\left( \frac{b}{a} \right)^{\frac{1}{3}}$

Equation (1): The vertical projection (A_v_) as measured by *Z_1_*:

$A_{v}=\pi ab$= $\pi\left( \frac{a}{b} \right)b^{2}$= $\pi\left( \frac{a}{b} \right)\left( \left( \frac{3*vol}{4*\pi} \right)^{\frac{1}{3}}*\left( \frac{b}{a} \right)^{\frac{1}{3}} \right)^{2}=\pi\left( \left( \frac{3*vol}{4*\pi} \right)^{\frac{1}{3}} \right)^{2}*\left( \frac{a}{b} \right)\left( \frac{b}{a} \right)^{\frac{2}{3}}$

= $\pi\left( \frac{3*vol}{4*\pi} \right)^{\frac{2}{3}}*\left( \frac{a}{b} \right)^{\frac{1}{3}}$ i.e. for fixed volume cell, A­_V_ scales with (a/b)^1/3^

Similarly, for Equation (2): the horizonal projection A*_h_*:

$A_{h}=\pi bc=\pi b^{2}$= $\pi\left( \left( \frac{3*vol}{4*\pi} \right)^{\frac{1}{3}}*\left( \frac{b}{a} \right)^{\frac{1}{3}} \right)^{2}=\pi\left( \left( \frac{3*vol}{4*\pi} \right)^{\frac{1}{3}} \right)^{2}*\left( \frac{b}{a} \right)^{\frac{2}{3}}$

i.e. for fixed volume, A­_H_ scales with (*b/a*)^2/3^

*Z_1_/Z_2_* = A_v_/A_h_ = = $\pi\left( \frac{3*vol}{4*\pi} \right)^{\frac{2}{3}}*\left( \frac{a}{b} \right)^{\frac{1}{3}}$/ $\pi\left( \frac{3*vol}{4*\pi} \right)^{\frac{2}{3}}*\left( \frac{b}{a} \right)^{\frac{2}{3}}$ which simplifies to (*a/b*); i.e. the linear trend in fig 2(e) is expected
